# T-Cell Regulation in Lepromatous Leprosy

**DOI:** 10.1371/journal.pntd.0002773

**Published:** 2014-04-10

**Authors:** Kidist Bobosha, Louis Wilson, Krista E. van Meijgaarden, Yonas Bekele, Martha Zewdie, Jolien J. van der Ploeg- van Schip, Markos Abebe, Jemal Hussein, Saraswoti Khadge, Kapil D. Neupane, Deanna A. Hagge, Ekaterina S. Jordanova, Abraham Aseffa, Tom H. M. Ottenhoff, Annemieke Geluk

**Affiliations:** 1 The Dept. of Infectious Diseases, Leiden University Medical Center, Leiden, The Netherlands; 2 Armauer Hansen Research Institute and ALERT hospital, Addis Ababa, Ethiopia; 3 Mycobacterial Research Laboratory, Anandaban Hospital, Kathmandu, Nepal; 4 The Dept. of Obstetrics and Gynaecology, Free University Amsterdam, Center for Gynaecologic Oncology, Amsterdam, The Netherlands; University of California San Diego School of Medicine, United States of America

## Abstract

Regulatory T (T_reg_) cells are known for their role in maintaining self-tolerance and balancing immune reactions in autoimmune diseases and chronic infections. However, regulatory mechanisms can also lead to prolonged survival of pathogens in chronic infections like leprosy and tuberculosis (TB). Despite high humoral responses against *Mycobacterium leprae* (*M. leprae*), lepromatous leprosy (LL) patients have the characteristic inability to generate T helper 1 (Th1) responses against the bacterium. In this study, we investigated the unresponsiveness to *M. leprae* in peripheral blood mononuclear cells (PBMC) of LL patients by analysis of IFN-γ responses to *M. leprae* before and after depletion of CD25^+^ cells, by cell subsets analysis of PBMC and by immunohistochemistry of patients' skin lesions. Depletion of CD25^+^ cells from total PBMC identified two groups of LL patients: 7/18 (38.8%) gained *in vitro* responsiveness towards *M. leprae* after depletion of CD25^+^ cells, which was reversed to *M. leprae*-specific T-cell unresponsiveness by addition of autologous CD25^+^ cells. In contrast, 11/18 (61.1%) remained anergic in the absence of CD25^+^ T-cells. For both groups mitogen-induced IFN-γ was, however, not affected by depletion of CD25^+^ cells. In *M. leprae* responding healthy controls, treated lepromatous leprosy (LL) and borderline tuberculoid leprosy (BT) patients, depletion of CD25^+^ cells only slightly increased the IFN-γ response. Furthermore, cell subset analysis showed significantly higher (p = 0.02) numbers of FoxP3^+^ CD8^+^CD25^+^ T-cells in LL compared to BT patients, whereas confocal microscopy of skin biopsies revealed increased numbers of CD68^+^CD163^+^ as well as FoxP3^+^ cells in lesions of LL compared to tuberculoid and borderline tuberculoid leprosy (TT/BT) lesions. Thus, these data show that CD25^+^ T_reg_ cells play a role in *M. leprae*-Th1 unresponsiveness in LL.

## Author Summary

Leprosy is a curable infectious disease caused by *Mycobacterium leprae* (*M. leprae*) that affects the skin and peripheral nerves. It is manifested in different forms ranging from self-healing, tuberculoid leprosy (TT) with low bacillary load and high cellular immunity against *M. leprae*, to lepromatous leprosy (LL) with high bacillary load and high antibody titers to *M. leprae* antigens. However, LL patients have poor cell mediated response against *M. leprae* leading to delayed clearance of the bacilli. A possible explanation for this bacterial persistence could lie in the presence of more regulatory cells at infection sites and in peripheral blood. This study shows the recovery of the cell mediated response by depletion of CD25^+^ cells in a subset of LL patients, while another patient subset was not affected similarly. Moreover, an increased frequency of FoxP3^+^ T cells together with anti-inflammatory macrophages was observed in LL patients' skin biopsies. Thus, these data show that CD25^+^ T_reg_ cells play a role in *M. leprae*-unresponsiveness in leprosy patients.

## Introduction

The human immune system strives to maintain the delicate balance between preventing host susceptibility to various pathogens and limiting immunopathology due to an exacerbated immune response to infections. Sub-populations of T-cells previously identified as suppressor T-cells and later as T_reg_ cells are the major players in the regulatory network of the immune system [1], [2]. Although the idea of suppressor T-cells was a key topic of research already in the 70's and 80's it was not successfully established because of poor cellular characterization, and it took until mid-1990's before T_reg_ cells were recognized as a different lineage [1]. More recently, studies clearly demonstrated the suppressive ability of this sub-population contributing to the re-acceptance of suppressor T-cell as a different T-cell lineage [3], [4].

Characterization of this T-cell sub-population has continued and currently the thymus-derived T_reg_ cells (tT_reg_ cells) and peripherally derived T_reg_ cells (pT_reg_ cells) [5] are the two widely accepted categories of T_reg_ cells [1], [6], [7]. Both T-cell subtypes play a role in limiting immune reactions in autoimmune diseases and chronic infections [8]–[11]. In addition, CD39^+^ T_reg_ cells have also been reported as a subset of the CD4^+^ CD25^high^FoxP3^+^ T_reg_ cells in association with chronic infections like tuberculosis (TB) [12], hepatitis B (HBV) and in graft rejections [13], [14] and the ability of CD8^+^ CD39^+^ T_reg_ cells to suppress antigen specific CD4^+^ proliferation clearly demonstrated the importance of this sub-population [15].

Leprosy is a chronic infectious disease leading to more than 200,000 new cases every year [16]. The remarkable inter-individual variability in clinical manifestations of leprosy closely parallels the hosts' abilities to mount effective immune responses to *M. leprae*. This is clear from the well-known immunological and clinical spectrum in those who progress to disease ranging from polar T helper 1 (Th1) to Th2 responses. TT and BT show more dominant Th1 responses which limit *M. leprae* growth resulting in clinical paucibacillary (PB) leprosy whereas, BL/LL patients demonstrate dominant Th2 responses as well as more permissive growth of *M. leprae* resulting in clinical multibacillary (MB) leprosy. TT/BT patients in general show high cellular responses and low antibody titers to *M. leprae* antigens, and develop localized granuloma with often no detectable bacilli in their lesions. The LL/BL patients at the opposite pole are incapable to generate *M. leprae* specific Th1 cell responses, show high antibody titers to *M. leprae* antigens, and poor granuloma formation with numerous bacilli in their lesions. The borderline states of leprosy are immunologically unstable. The different outcomes of infection in leprosy are most likely caused by host defense mechanisms [17]. However, the mechanism underlying the *M. leprae*-specific T-cell anergy in LL patients is still not completely understood.

In chronic bacterial or viral infections, evidence exists that T_reg_ cells suppress effector T-cells (T_eff_ cells) in order to limit damage to the host caused by the immune responses against pathogens [18]. In this situation, the regulatory activity of T_reg_ cells may lead to prolonged survival of pathogens in the host [9], [19]. As evidenced in a previous study, higher levels of CD4^+^CD25^+^FoxP3^+^ T_reg_ cells were observed in active TB patients in the periphery compared to latently infected individuals and healthy controls [20], [21]. Also, an increased number of T_reg_ cells expressing FoxP3, cytotoxic T-lymphocyte antigen 4 (CTLA-4) and glucocorticoid-induced tumour-necrosis-factor-receptor-related protein (GITR) were reported in lymphnodes from children with tuberculosis lymphadenitis [22]. Similarly, in leprosy, higher numbers of T_reg_ cells in PBMC from BL and LL patients stimulated with *M. leprae* cell wall antigen (MLCWA) were observed compared to TT/BT forms, indicating the possibility that T_reg_ cells may have a role in persistence of *M. leprae* bacteria as well as unresponsiveness of Th1 cells in BL/LL patients [23]. Recently, the mechanism of action of FoxP3 in CD4^+^CD25^+^ T cells derived from BL/LL leprosy patients was shown to result from increased molecular interactions of FoxP3 with Histone deacetylases (HDAC7/9) in the nucleus of CD4^+^CD25^+^ T cells derived from BL/LL patients [24].

In the presence of pathogens, T_reg_ cells can also be induced by certain macrophages as evidenced by the anti-inflammatory, CD163^+^ macrophages, known as type 2 macrophages (mφ2), that exert a suppressive effect on Th1 responses [25], [26]. On the other hand, IL-10 induced phagocytosis of *M. leprae* by mφ2 without induction of microbicidal activity in LL lesions has been described [27] indicating the role of IL-10 producing T_reg_ cells in the persistence of the pathogen within the host. Similarly, the presence of higher IL-10 expression correlated with increased CD163 and indoleamine 2,3-dioxygenase (IDO) proteins in tissues and sera of LL patients further evidenced their potential [28].

In this study, we have investigated the functional role of CD25^+^ T_reg_ cells in *M. leprae* unresponsiveness of LL patients as well as the frequency of CD25^+^ and FoxP3^+^ cells in the PBMC of leprosy patients. Additionally, lesions of LL and TT/BT patients were assessed for the presence of FoxP3^+^ cells and CD163^+^ macrophages (mφ2).

## Materials and Methods

### Ethical statement

Ethical approval of the study protocol was obtained from the National Health Research Ethical Review committee, Ethiopia (NERC # RDHE/127-83/08) and the Nepal Health Research Council (NHRC #751). Participants were informed about the study objectives, the required amount and kind of samples and their right to refuse to take part or withdraw from the study at anytime without consequences for their treatment. Written and Informed consent was obtained from study participants before enrollment.

### Study participants

The following HIV-negative individuals were recruited on a voluntary basis: newly diagnosed, non reactional leprosy patients from Ethiopia (ALERT hospital, Addis Ababa, Ethiopia) classified as LL (n = 40) and TT/BT (n = 16) and healthy endemic controls from health centers in Addis Ababa (EC; n = 5); Treated, non reactional LL (n = 6) and TT/BT (n = 9) patients and EC (n = 10) from Anandaban Hospital, (Kathmandu, Nepal); and non-endemic Dutch healthy controls (NEC; n = 13). Leprosy was diagnosed based on clinical, bacteriological and histological observations and classified by a skin biopsy evaluated according to the Ridley and Jopling classification [17] by qualified microbiologists and pathologists. All patients were enrolled before treatment was initiated. EC were assessed for the absence of clinical signs and symptoms of tuberculosis and leprosy. Individuals working in health facilities were excluded as EC.

### PBMC isolation, freezing and thawing

PBMC were isolated by Ficoll-Hypaque density gradient method, cells were washed and suspended in 20% fetal calf serum (FCS) in AIM-V (Invitrogen, Carlsbad, CA) and kept cool on ice, counted and frozen using a cold freshly prepared freezing medium composed of 20% FCS, 20% dimethyl sulphoxide (DMSO) in AIM-V. Cells were kept at −80°C for 2–3 days and transferred to liquid nitrogen until use. During thawing, cells were transported in liquid nitrogen to a water bath (37°C) for 30 to 40 seconds until thawed half way and resuspended in 10% FCS in AIM-V (37°C) containing 1/10,000 benzonase until completely thawed, washed 2 times (5–7 minutes each) and counted. The percentage viability obtained was >75% and cells were incubated with anti-CD25 magnetic beads or used for FACS analysis.

### CD25 ^+^cell separation

Frozen PBMC were thawed, washed and incubated with 20 µl of the CD25 micro beads II, human (Miteny Biotec, Bergisch Gladbach, Germany) in 80 µl MACS buffer (Phosphate-buffered saline (PBS) with 0.5% Bovine serum albumin (BSA) and 2 mM EDTA) for 20 minutes at 4°C. Cells were washed and added to MS column attached to Magnetic Cell Sorter (MACS) (Milteny Biotec) where CD25^−^ cells were collected as flow through and the CD25^+^ population was collected by detaching the column from the magnetic cell sorter. Cells were washed with MACS buffer and resuspended in AIM-V medium. The purity of the CD25^−^ and CD25^+^cell populations was >80% (supplementary figure S2A and S2B).

### Lymphocyte stimulation tests (LST)

Total PBMC (150,000 cells/well), CD25^−^ cells (150,000 cells/well) or CD25^−^ cells with proportionally added CD25^+^ cells (10,000 and/or 25,000) were added in triplicate into 96 well U bottom tissue culture plates and cultured with *M. leprae* whole cell sonicate (WCS; 10 µg/ml), phytohaemagglutinin (PHA; 1 µg/ml) or AIM-V medium at 37°C with 5% CO_2_ and 70% humidity. After 6 days, supernatants were collected and kept frozen until used in ELISA.

### *M. leprae* whole cell sonicate (WCS)

Irradiated armadillo-derived *M. leprae* whole cells were probe sonicated with a Sanyo sonicator to >95% breakage. This material was kindly provided by Dr. J.S. Spencer through the NIH/NIAID “Leprosy Research Support” Contract N01 AI-25469 from Colorado State University (now available through the Biodefense and Emerging Infections Research Resources Repository listed at (http://www.beiresources.org/TBVTRMResearch Materials/tabid/1431/Default.aspx).

### IFN-γ ELISA

IFN-γ levels were determined by ELISA (U-CyTech, Utrecht, The Netherlands) [29]. The cut-off value to define positive responses was set beforehand at100 pg/ml. The assay sensitivity level was 40 pg/ml. Values for unstimulated cell cultures were typically <40 pg/ml.

### Flow cytometry

After depletion, the total PBMC, CD25^−^ or CD25^+^ populations (25,000 to 200,000 cells) were stained for CD3 (clone SK7, PerCP; Becton, Dickinson and Company, New Jersey, USA), CD4 (clone SK3, FITC; BD) and CD25 (PE; MACS) to check the purity.

Frozen PBMC of patients and healthy controls (2×10^6^ cells/ml) were thawed, washed and treated with benzonase (10 U/ml, Novagen, Merck4Biosciences, Merck KGaA, Darmstadt, Germany) for 2 hours prior to *in vitro* stimulation with PMA (20 ng/ml)/ionomycine (500 ng/ml) in the presence of 1 µg/ml anti CD28 (Sanquin, the Netherlands) and 1 µg/ml anti CD49d (BD Biosciences, Eerbodegem, Belgium). After 4 hours, Brefeldin A (Sigma Aldrich) was added at 3 µg/ml and cells were left for an additional 16 hours in the incubator at 37°C with 5% CO_2_ and 70% humidity. After live/dead staining with Vivid (Invitrogen, Life technologies, Merelbeke, Belgium), surface staining was performed for 30 minutes at 4°C with the labeled antibodies directed against: CD14- and CD19-Pacific Blue, CD3-PE-TexasRed (all Invitrogen, Life technologies), CD8-Horizon V500, CD4-Pe-Cy7, CD25-APC-H7 (all BD Biosciences), and CD39-PE (Biolegend, ITK Diagnostics, Uithoorn, The Netherlands). Samples were washed, fixed and intracellular staining was performed using the intrastain kit (Dako Diagnostics, Glostrup, Denmark) with IFN-γ -Alexa700 (BD Biosciences), IL-10 APC (Miltenyi Biotec GmbH, Bergisch Gladbach, Germany), and FoxP3 PE-Cy5 (eBioscience, Hatfield, UK) labeled antibodies. Cells were acquired on a FACS LSR Fortessa with Diva software (BD Biosciences, The Netherlands) and analyzed with FlowJo version 9.4.1 (Tree Star, Ashland, OR, USA). The full gating strategy for live CD4^+^ CD3^+^ cells or CD8^+^ CD3^+^ cells (supplementary Figure S1A and S1B) was performed in compliance with the most recent MIATA [30] guidelines according to the following procedure: events were first gated using a forward scatter area (FSC-A) versus height (FSC-H) plot to remove doublets. Subsequently, the events were subjected to a lymphocyte gate using a side scatter (SSC) followed by a live/dead gating. Then, live CD3^+^ cells were gated and CD14^+^ and CD19^+^ events were excluded from analysis using a dump channel. Finally, CD3 live cells were separated in to CD4^+^ and CD8^+^. After the gates for each function were created, we used the Boolean gate platform to identify all functions within each cell preparation using the full array of possible combinations.

### Immunohistochemistry and confocal microscopy

Skin biopsies taken from leprosy lesions of LL (n = 10) and TT/BT (n = 4) patients were fixed in formalin and embedded in paraffin. Tissue sections with 4 µm thickness were prepared using a microtome (LEICA RM 2165). The prepared tissues sections were stained for hematoxylin and eosine (H & E; images are shown in supplementary figure S3) and also used as previously described [31] for immunofluorescence staining. Tissue sections were deparaffinised and rehydrated using graded concentrations of ethanol to distilled water. Antigen retrieval was performed in boiling Tris-EDTA buffer (10 mM Tris Base, 1 mM EDTA Solution, 0.05% Tween 20, pH 9.0) for 12 minutes. After two hours of cooling at room temperature in antigen retrieval buffer, slides were washed twice in distilled water and twice in PBS, blocked for 15 min with 5% goat serum in PBS, washed again with PBS and stained with primary antibodies for FoxP3 (1∶100, mouse anti-human IgG1 Abcam; Cambridge, UK), CD8 (1∶100 mouse anti-human IgG2b, Abcam), CD68 (mouse anti-human IgG2a AbD serotec/Bio-Rad; Veenendaal, The Netherlands), CD163 (1∶400, mouse anti-human IgG1, Leica; Rijswijk, The Netherlands) and CD39 (1∶100, mouse anti-human IgG2a, Abcam). Two antibodies were used per tissue section: FoxP3 with CD68, CD163, CD39 or CD8; CD68 with CD163 and CD39 with CD163. After overnight incubation at room temperature in the dark, sections were washed and incubated for 1 hour in the dark with secondary antibodies; goat-anti-mouse IgG1 coupled with Alexa 488 (1∶200) (Invitrogen,Bleiswijk The Netherlands), goat-anti-mouse IgG2a or goat-anti-mouse IgG2b with Alexa 546 (1∶200) (Invitrogen). Tissue sections were then washed three times with PBS and mounted with Vectashield (DAPI, 4′, 6-diamidino-2-phenylindole; Vector Laboratories, Brussels, Belgium). Immunofluorescence of skin sections was examined and images were taken from 5 different fields per section using a Leica-TCS-SP5 confocal laser scanning microscope (Leica Microsystems, Mannheim, Germany). Nucleated cells that positively stained for the specific marker were counted from five different fields per section by two laboratory persons independently. Average counts for each marker per section were compared for all samples.

### Statistical analysis

Differences in cytokine concentrations were analyzed with the two-tailed Mann-Whitney U test or Wilcoxon signed rank test for non-parametric distribution using GraphPad Prism version 5.01 for Windows (GraphPad Software, San Diego California USA; www.graphpad.com) P-values were corrected for multiple comparisons. The statistical significance level used was p<0.05.

## Results

### Depletion of CD25^+^ cells enhanced pro-inflammatory response in LL patients

To analyse the role of CD25^+^ cells in the production of IFN-γ, PBMC from Ethiopian LL patients (n = 17) and Dutch healthy controls (n = 12) were depleted of CD25^+^ cells and cell subsets with and without re-added CD25^+^ cells were stimulated with *M. leprae* WCS in 6 days culture.

**Figure 1:**
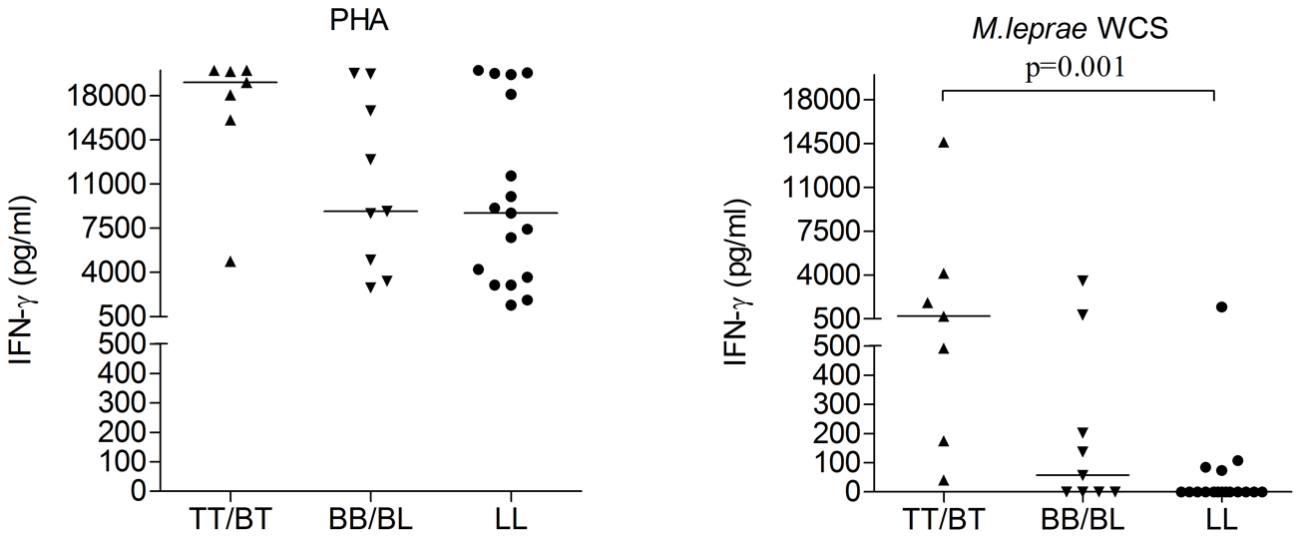
IFN-γ responses to PHA and *M. leprae* whole cell sonicate (WCS) by PBMC of TT/BT (n = 7), BB/BL (n = 9) and LL (n = 16) patients. Median values for each group are indicated by horizontal lines.

PBMC from treated Nepali LL (n = 6), BT (n = 9) patients and EC (n = 10) were depleted for CD25^+^ cells but only the total PBMC and CD25^−^cell subset were stimulated with *M. leprae* WCS. When compared according to clinical classification, there was a trend of higher IFN-γ production in PB compared to MB samples. IFN-γ production of total PBMC (undepleted fraction) from LL patients in response to *M. leprae* (WCS) was significantly lower (p = 0.001) compared to responses by PBMC from TT/BT patients, whereas IFN-γ responses to PHA were high in both groups (Fig. 1). These data further confirm the *M. leprae*-specific lack of cell mediated immunity (CMI) in LL patients.

**Figure 2:**
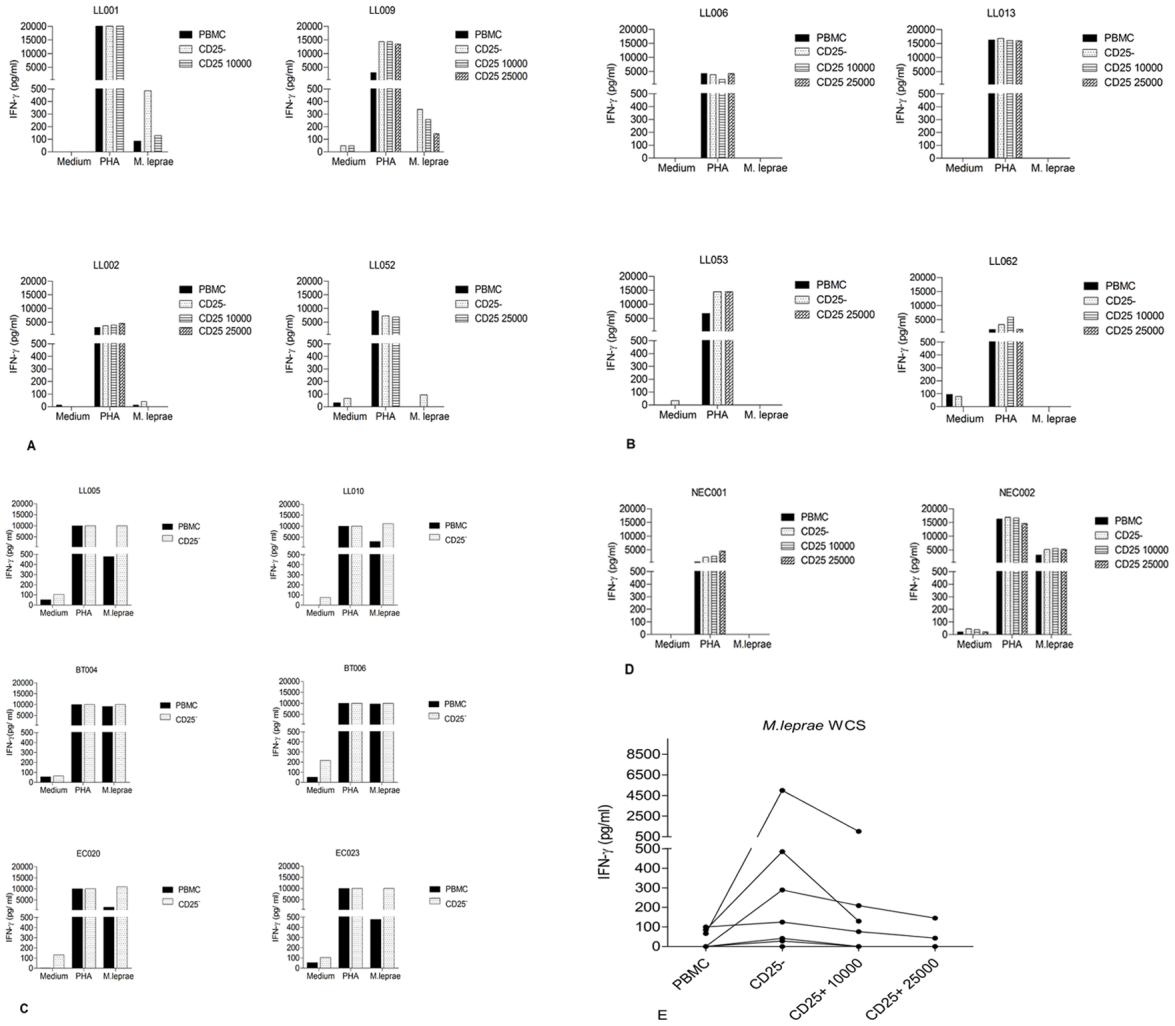
IFN-γ responses of total PBMC, CD25^−^ cells and CD25^−^ cells supplemented with CD25^+^ cells from LL patients. (**A**) representatives for the group responding to *M. leprae* after depletion of CD25^+^ cells (n = 7); (**B**) representatives for the group not responding to *M. leprae* after depletion of CD25^+^ cells (n = 11); (**C**) LL005 and LL010 representatives for Nepali treated LL patients (n = 10), BT004 and BT006 representatives for Nepali treated BT patients (n = 7) and EC020 and EC023 representatives for Nepali EC (n = 10) before and after depletion of CD25^+^ cells; (**D**) NEC001 and NEC002 representatives for healthy Dutch controls (n = 10) after depletion of CD25^+^ cells with and without response to *M. leprae* WCS; (**E**) Dot plot graph showing IFN-γ responses of both groups of Ethiopian LL patients in dot-plot graph. Medium indicates AIM-V medium used in the assays as negative control. In 2A and 2B: for LL001, CD25-25000 and for LL052 and LL053, CD25-10000 were not done.

Analysis of IFN-γ production in response to *M. leprae* (WCS) by CD25^−^ cells alone or CD25^−^ cells (150,000 cells per well) supplemented with the CD25^+^ fraction (10,000 or 25,000 cells/well) discriminated two groups of LL patients: those that produced IFN-γ in response to *M. leprae* after CD25^+^ cell depletion and those that did not (Fig. 2A, 2B and 2E). Among the 18 LL Ethiopian patients, 7 (38%) responded to *M. leprae* WCS after depletion of CD25^+^ cells whereas they lacked any response in total PBMC. IFN-γ production in response to PHA in both groups was not affected by the depletion of or enrichment with CD25^+^ cells.

In the LL patient group, in which recovery of IFN-γ responses was observed to *M. leprae* WCS after depletion of CD25^+^ cells, this could be reversed proportionally by the addition of CD25^+^ cells (Fig. 2A). In the patient group in which CD25^+^ cell depletion did not reverse anergy to *M. leprae*, there was no effect observed by addition of CD25^+^ cells to the depleted fraction (Fig. 2B).

In similar analysis of treated leprosy patients (LL and BT) and endemic controls from a Nepali population, PBMC responded to *M. leprae* WCS in the presence of CD25^+^ cells and a slight increase in IFN- γ levels after CD25^+^ cell depletion was also observed (Fig. 2C). Similarly, healthy Dutch controls (n = 8) responding to *M. leprae* WCS before depletion of CD25^+^ cell showed a slight increase after depletion (Fig. 2D left panel) as well, while other NEC (n = 5) remained unresponsive after CD25^+^ cell depletion (Fig. 2D right panel).

### FoxP3 expressing CD8^+^ CD25^+^ T-cell are more abundant in PBMC of LL

**Figure 3:**
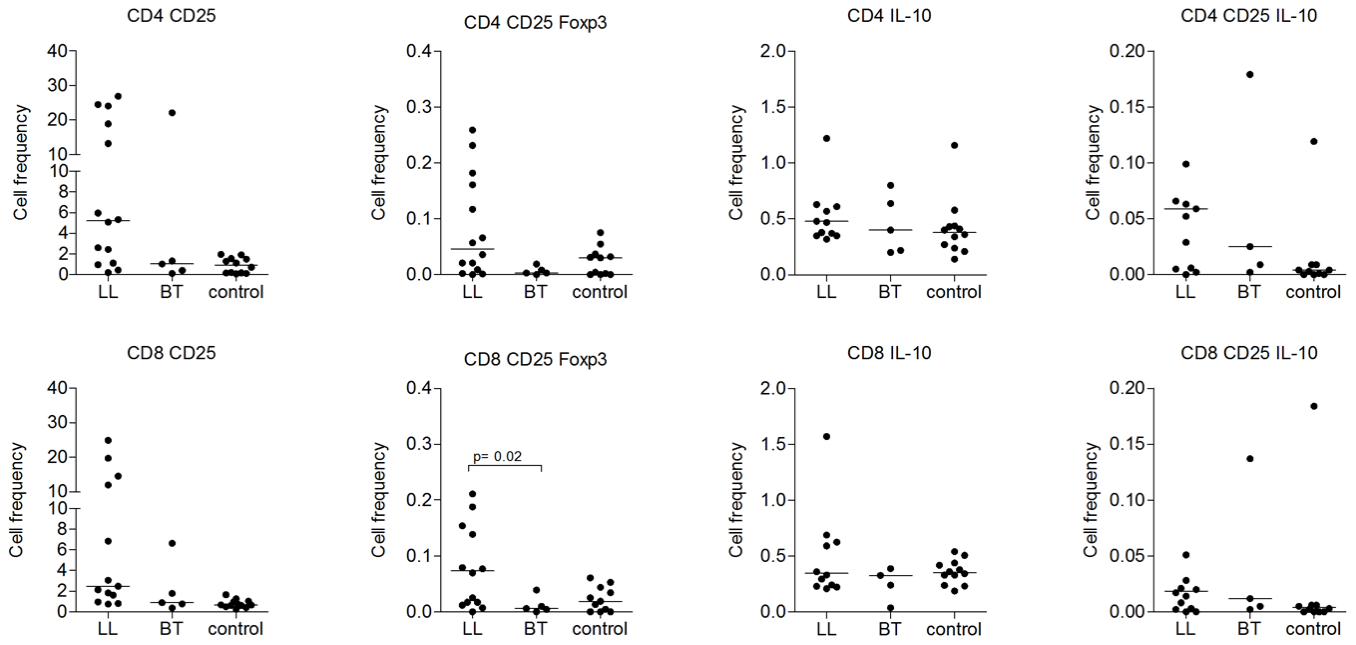
T-cell subset analysis of PBMC from LL, TT/BT and the control group consisting of EC and NEC showing the frequencies of FoxP3 expressing T-cells and IL-10 producing FoxP3^+^ T-cells.

For cell subset analysis, PBMC from Ethiopian LL (n = 13), TT/BT (n = 5) and EC (n = 7) and Dutch healthy controls (NEC; n = 4) were stained for surface and intra-cellular markers. The frequency of FoxP3^+^ CD8^+^CD25^+^ cells was significantly higher in PBMC of LL patients compared to TT/BT patients (p = 0.02) (Fig. 3). Although not statistically significant (p = 0.05), we also observed a higher frequencies of FoxP3^+^ CD4^+^ CD25^+^ T-cell in the LL group compared to the TT/BT patients (Fig. 3). In contrast, analysis of the frequency of IL-10 producing CD4^+^ CD25^+^ or CD8^+^CD25^+^ T-cell showed no significant differences between patients and healthy controls. The frequency of IL-10 production in CD4^+^ CD25^+^ or CD8^+^CD25^+^ T-cell in general was very low in all groups.

### Mφ2 (CD68^+^ CD163^+^) and FoxP3^+^ cells are more frequent in skin lesions of LL patients

**Figure 4:**
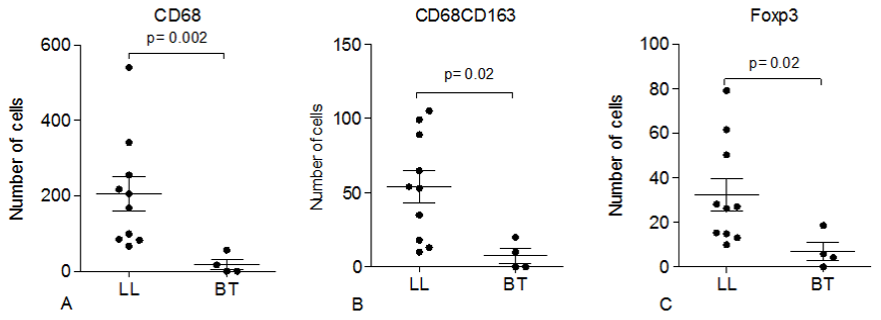
Immunohistochemical analysis of skin lesions of LL (n = 10) and BT patients (n = 4) showing the number of (A) CD68^+^ cells (B) CD68^+^ CD163^+^ cells and (C) FoxP3^+^ cells.

**Figure 5:**
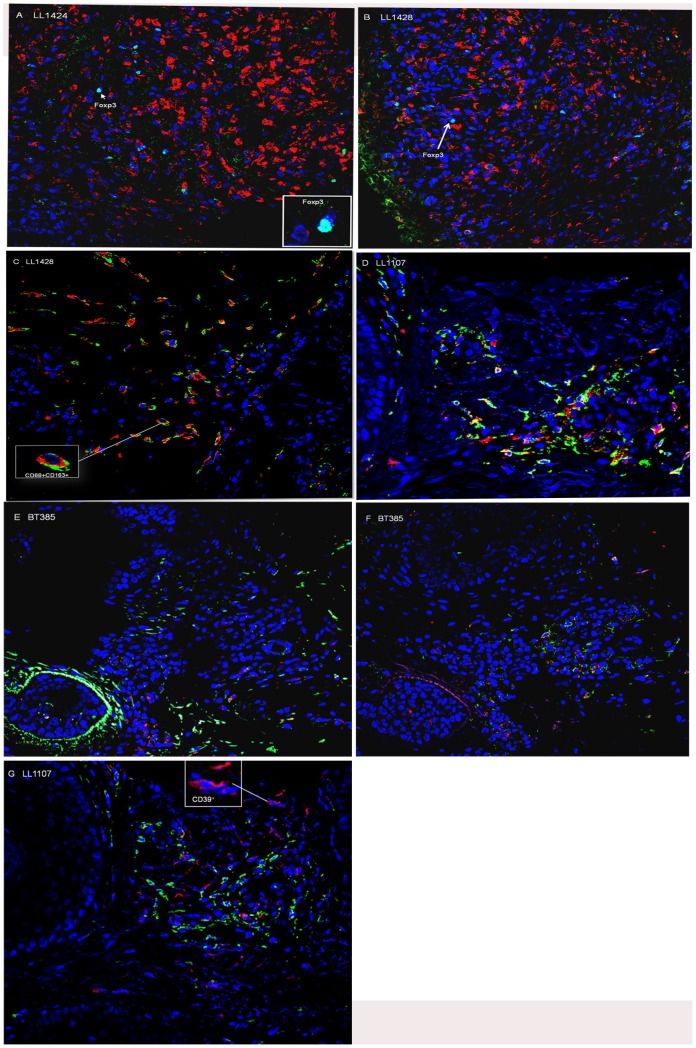
Immunohistochemical analysis (Original magnification, 400×; image size 359 µm×359 µm) of skin lesions. Sequential skin sections from LL (n = 10) and BT (n = 4) patients were stained with mAb specific for CD68 (red) and FoxP3 (green) [**A**, **B**, **E**, **F**], for CD68 (red) and CD163 (green) [**C**, **D**] and CD39 (red) [**G**]. Representatives LL [**A**, **B**, **C**, **D**, **and** **G**] and BT [**E**, **F**,] patients are shown. Insets represent 1500× magnification of FoxP3^+^ cells [**A**, **B**]; 800× magnification of CD68^+^ CD163^+^ [**C**, **D**]; 1000× magnification of CD39^+^ cells [**G**].

Confocal analysis of two-colour immunofluorescence was used to localize specific cell markers in skin biopsies of Ethiopian LL (n = 10) and TT/BT (n = 4) leprosy patients. Higher number of CD68^+^ cells in LL lesions (p = 0.02) (Fig. 4A, 5A and B) indicated the presence of more infiltrating macrophages compared to TT/BT (Fig. 5C and D). In addition, CD68^+^ CD163^+^ cells (mφ2) and FoxP3^+^ cells were present to a larger extent in LL patients' lesions (p = 0.02) compared to TT/BT (Fig. 4B, 4C, 5C and 5D). With respect to the numbers of CD68^+^ CD163^+^ cells (mφ2) and FoxP3^+^ cells, differences were observed among the LL patients which could be explained by variations in the time elapsed since skin lesions were noticeable or by influence of other host factors. Although we found significantly higher frequency of CD8^+^FoxP3^+^ in PBMC, we could not clearly detect CD8^+^FoxP3^+^ in the skin lesions indicating CD4^+^FoxP3^+^ cells could play a regulatory role in these tissues. In addition, skin lesions were stained with CD39 combined with FoxP3 to localize CD39^+^FoxP3^+^ regulatory T-cells. However, in most skin tissues, CD39^+^ cells were not detected except for two LL skin tissues in which CD39 and FoxP3 positivity was observed simultaneously in macrophage-like shaped cells (Fig. 4E). Thus, these results indicate the induction of more FoxP3^+^ but not CD39^+^ Treg cells in LL patients' skin lesions probably by the presence of type 2 macrophages.

## Discussion

Decreased *M. leprae*-specific T-cell mediated immunity is the hall mark of lepromatous multibacillary leprosy and can be assessed by *in vitro* unresponsiveness to *M. leprae* (antigens) or clonal anergy [2], [23], [32]. In this study, we confirm the *M. leprae*-specific unresponsiveness by the absence of IFN-γ responses to *M. leprae* WCS.

Several studies have investigated the possible causes leading to hyporesponsiveness in LL patients such as formation of foamy macrophages in presence of IL-10 [27], cholesterol dependent dismantling of HLA-DR raft in macrophages of BL/LL [33] and other factors, including T_reg_ cells. Some of these studies on T_reg_ cells have shown their presence and role either in the periphery or in skin lesions through measuring T_reg_ associated markers, mainly CD25, TGF-β, CTLA4, IL-10, and FoxP3 [23], [24], [34], [35]. Recently, Teles *et al.* showed higher expression of IFN-γ and the downstream vitamin D-dependent antimicrobial pathway related genes including CYP27B1 and VDR (Vitamin D receptor) in TT/BT as well as an increased IL-10 expression induced by IFN-β in LL lesions [36]. Some reports have revealed the limitations of the available T_reg_ markers due to their lack of specificity [37]–[39]: CD25, for example, is expressed on activated T and B cells and is not exclusively found on T_reg_ cells. However, noting that CD25 is still a crucial marker for T_reg_ cells in the unstimulated situation, we performed depletion of CD25^+^ cells from unstimulated PBMC to isolate the T_reg_ cells and demonstrated their involvement in *M. leprae*-specific unresponsiveness in LL patients.

The BL/LL patients are known for their poor CMI and this is commonly assessed by measuring IFN-γ responses to *M. leprae* WCS. The total PBMC of the LL patients were analysed along with the CD25^+^ depleted and enriched fraction for their IFN-γ responses to *M. leprae* WCS and was negative. However, the depletion of CD25^+^ cells from total PBMC of LL patients showed an enhanced pro-inflammatory response as measured by the level of IFN-γ in response to *M. leprae* WCS in some but not all patients. Two distinct groups of LL patients were identified after depletion of CD25^+^ cells; 38% (7/18) of the LL patients showed enhanced IFN-γ responses in the CD25^−^ population while the remaining 62% of the LL patients did not respond to *M. leprae* WCS at all. The recovered IFN-γ production in the first group was reversed by addition of CD25^+^ cells, clearly indicating that this CD25^+^ cell population conferred the unresponsiveness in these LL patients. However, we did not stain the CD25^+^ cell populations with FoxP3 which could have allowed more detailed characterization as CD25^high^ FoxP3 or CD25^low^ FoxP3 sub-populations which might have explained differences between the responders and non-responders. Nonetheless, the presence of non-responding LL patients after depletion of CD25^+^ cells indicates that CD25^+^ T_reg_ cells do not represent the sole factor responsible for T-cell anergy in LL leprosy. As the Th1 arm is responsible for killing and clearing bacilli, there could have been enormous damage to tissues in BL/LL patients where high load of bacilli and antigens are available. However, the presence of T_reg_ in these patients represents one important factor that can avoid tissue damage but, on the other hand, creates a convenient environment for bacilli to survive through suppression of Th1 response. In addition, the significant IFN-γ production observed in treated LL patients in our study before depletion of CD25^+^ T cells showed how treatment and thereby the level of bacillary load can influence the Th1 response and T_reg_. Similar findings were reported for TB patients with recovered IFN-γ production and reduced number of T_reg_ cells after treatment [21], [40]. The slight increases observed in IFN-γ production after depletion of CD25^+^ T cells in treated LL and BT patients and in EC tested in the depletion experiments could also indicate the regular presence of T_reg_ cells to maintain homeostasis in the host. However, the overall ratio of CD25^+^ T_reg_ cells to effector T cells will be crucial in determining the outcome of *M. leprae* infection in the host.

Previous studies which aimed at identifying potential factors for *M. leprae*-specific unresponsiveness in LL used the addition of IL-2 [2], [41]–[43] or anti-DQ monoclonal antibodies [44] or offered isolated antigenic fractions of *M. leprae*. Interestingly, each of the studies similarly identified two groups of LL patients, in one of which *M. leprae* unresponsiveness could be reversed. This indicated that the unresponsive phenotype in LL patients is likely mediated through the collective effects of various molecules. The more recent observation of cholesterol-dependent dismantling of HLA-DR raft and an increased membrane fluidity in BL/LL patients which causes a major defect in antigen presentation provides additional evidence for the presence of multiple different factors leading to T-cell anergy [33]. Thus, *M. leprae* specific unresponsiveness/anergy in LL patients very likely is a complex phenomenon mediated by multiple host and pathogen associated factors, one of which is represented by T_reg_ cells.

Several studies have reported on the *ex vivo* frequency of T_reg_ cells in peripheral blood of LL and TT/BT patients in unstimulated or *M. leprae* antigens stimulated PBMC [23], [35]. Attia *et al*. showed, elevated frequencies of circulating T_reg_ cells (CD4^+^CD25^high^FoxP3^+^) in TT patients [35] whereas Palermo *et al.*, showed that PBMC stimulated with *M. leprae* antigen for 6 days in culture had significantly higher number of T_reg_ cells (CD4^+^ CD25^+^FoxP3^+^) in LL patients [23]. Recently, Saini *et al.*, further confirmed the importance of T_regs_ in LL non-responsiveness by measuring TGF-*β* producing CD4^+^ CD25^+^FoxP3^+^ cells in stimulated PBMC culture [45]. In this study, we analysed the frequency of T_reg_ cells in PBMC briefly activated with PMA/ionomycin. The frequency of CD4^+^ CD25^+^FoxP3^+^ cells was higher in LL compared to BT but not statistically significant (Fig. 3). However, with the visible difference observed between LL and BT and with the evidences from previous studies, their presence and role in BL/LL patients cannot be denied. For example, the recent molecular analysis of FoxP3 in CD4^+^CD25^+^ T cells nuclei has revealed that the FoxP3 interaction with histone deacetylases drives the immune suppression by CD4^+^ CD25^+^ T_regs_ in BL/LL unlike in other forms of leprosy [24].

On the other hand, the frequency of CD8^+^ CD25^+^FoxP3^+^ cells found in this study was significantly higher in LL (Fig. 3). This suggests that FoxP3^+^ CD8^+^ CD25^+^ T_reg_ cells may also play a role in unresponsiveness in LL although not specifically analyzed for their functional role in our depletion experiments. Although lower in frequency compared to the CD4^+^ CD25^+^FoxP3^+^, Saini *et al.*, also reported higher numbers of CD8^+^ CD25^+^FoxP3^+^ in LL compared to BT but without induction of TGF-β [45]. Most studies focused on CD4^+^ CD25^+^FoxP3^+^ in leprosy [23], [35]. In contrast one study on LL lesions showed the presence of increased numbers of CD8^+^ T cells with suppressive type in LL indicating the importance of CD8^+^ T_reg_ cells in leprosy [46]. In addition few other studies identified CD8^+^ T_reg_ as a potential suppressive sub-population [47], [48]. Recent evidence from an *in vitro* study also revealed CD8^+^ T_reg_ cells (CD8^+^ LAG-3^+^ FoxP3^+^CTLA-4^+^) induced by matured plasmacytoid dendritic cells (pDC) with suppression activity on allo-reactive T memory cells [49]. In our opinion, the CD8^+^ T_reg_ population is not sufficiently studied in leprosy and we believe further analysis of this population in all forms of leprosy in periphery and lesionary tissues will be vital.

The low IL-10 frequency measured by FACS analysis in all groups did not allow detection of significant differences among groups as expected in view of the crucial role of IL-10 as an anti-inflammatory cytokine in the unresponsiveness in LL patients [27], [36]. This could be due to the short PMA/ionomycin stimulation inherent to the procedure for *ex vivo* determination of the frequency of CD25^+^ cells. However, 6 days stimulation of PBMC from BL patients with *M. leprae* induced high levels of IL-10 [50].

Although, it will not be easy to generalize or conclude on frequencies and numbers of CD4^+^ CD25^+^FoxP3^+^ T_reg_ cells in different forms of leprosy since the experimental procedures used in each study vary, most of the studies including ours, point to the presence of increased numbers of T_reg_ cells in LL patients either in periphery as well as lesions. Detailed characterization of T_reg_ cell subsets in large cohorts of leprosy patients as well as the ratio to effector T cells may provide additional insights in this area.

The dominant presence of CD163^+^ macrophages in LL lesions [27], [28] and the significantly higher expression of IL-10 and CTLA4 in LL tissues have been reported previously [25]. The role of T_reg_ cells (FoxP3^+^ GITR^+^ CD25^+^) and their induction by CD163^+^ anti-inflammatory human macrophages was demonstrated *in vitro* since CD4^+^ T-cells gained a potent regulatory/suppressor phenotype and functions after activation by mφ2 [25]. In the current study, we show the presence of significantly higher number of CD68^+^ CD163^+^cells (mφ2) in the vicinity of FoxP3^+^ cells in LL lesions compared to TT/BT lesions. These findings support the involvement of both cell types in the induction and/or maintenance of *M. leprae* directed T_reg_ cells in LL lesions.

Since a suppressive effect of CD4^+^CD39^+^FoxP3^+^ T_reg_ cells was described in TB patients [12], we also analysed the frequency of CD39^+^FoxP3^+^ cells in PBMC but observed no differences between LL and TT/BT patients except for few LL skin lesions, in which macrophage-shaped CD39^+^ cells were observed. A recent study has shown that CD39 expression on macrophages has an important role in self-regulation mechanism during inflammation [51]. These cells may also play a similar role in LL patients but this has to be further analysed.

In summary, this study clearly show that CD25^+^ T_reg_ cells play a role in unresponsiveness in LL, and that there are two subtypes of *M. leprae* unresponsive LL patients. Furthermore, the co-existence of T_reg_ cells with mφ2 in LL lesions further supports the potential role of these regulatory cell subsets at the site of infection.

## Supporting Information

Figure S1 **A. Gating strategy for live CD4^+^CD3^+^ cells or CD8^+^CD3^+^ cells in PBMC.** Ungated events were first gated using a forward scatter area (FSC-A) versus height (FSC-H) plot to remove doublets. Subsequently, the events were subjected to a lymphocyte gate by gated through a side scatter (SSC). Subsequently, live CD3^+^cells were gated by live/dead staining using Vivid (Invitrogen, Life technologies) as a marker for viability and CD14^+^ or CD19^+^ events were excluded from analysis using a dump channel. Finally, CD3^+^ live cells were separated into CD4^+^ and CD8^+^. **B. Gating strategy for IL-10 and FoxP3 expression in CD4^+^CD3^+^ cells or CD8^+^CD3^+^ cells.** After the gates for each function were created, we used the Boolean gate platform to identify all functions within each cell population using the full array of possible combinations FACS LSR Fortessa as shown here for IL-10 and FoxP3 expression in CD4^+^ T cells.
(TIF)

Figure S2 **A. Dot plot analysis of bulk (total) PBMC, CD25 depleted and CD25 positive population of a representative LL patient (LL053).** After separating the CD25 negative and CD25 positive cell population using Magnetic cell sorter, fractions of each cell population including the bulk (total) PBMC were analysed for their expression of CD3, CD4 and CD25. Here the data are presented in dot plots. **B. Zebra plots of bulk (total) PBMC, CD25 depleted and CD25 positive population of a representative LL patient (LL053).** After separating the CD25 negative and CD25 positive cell population using Magnetic cell sorter, fractions of each cell population including the bulk (total) PBMC were analysed for their expression of CD3, CD4 and CD25. Here the data are presented in zebra plots.
(TIF)

Figure S3 **Hematoxylin and Eosin staining of four representative LL patients (original magnification ×100).** Tissue sections from paraffin embedded biopsy samples of leprosy patients were stained for H&E. Here images of H&E staining of four representative LL patients are presented.
(TIF)
