# Supplementary figures and images for: T-Cell Regulation in Lepromatous Leprosy
Source: PLoS Negl Trop Dis. 2014 Apr 10;8(4):e2773. doi: 10.1371/journal.pntd.0002773 (PMC3983090; doi:10.1371/journal.pntd.0002773)

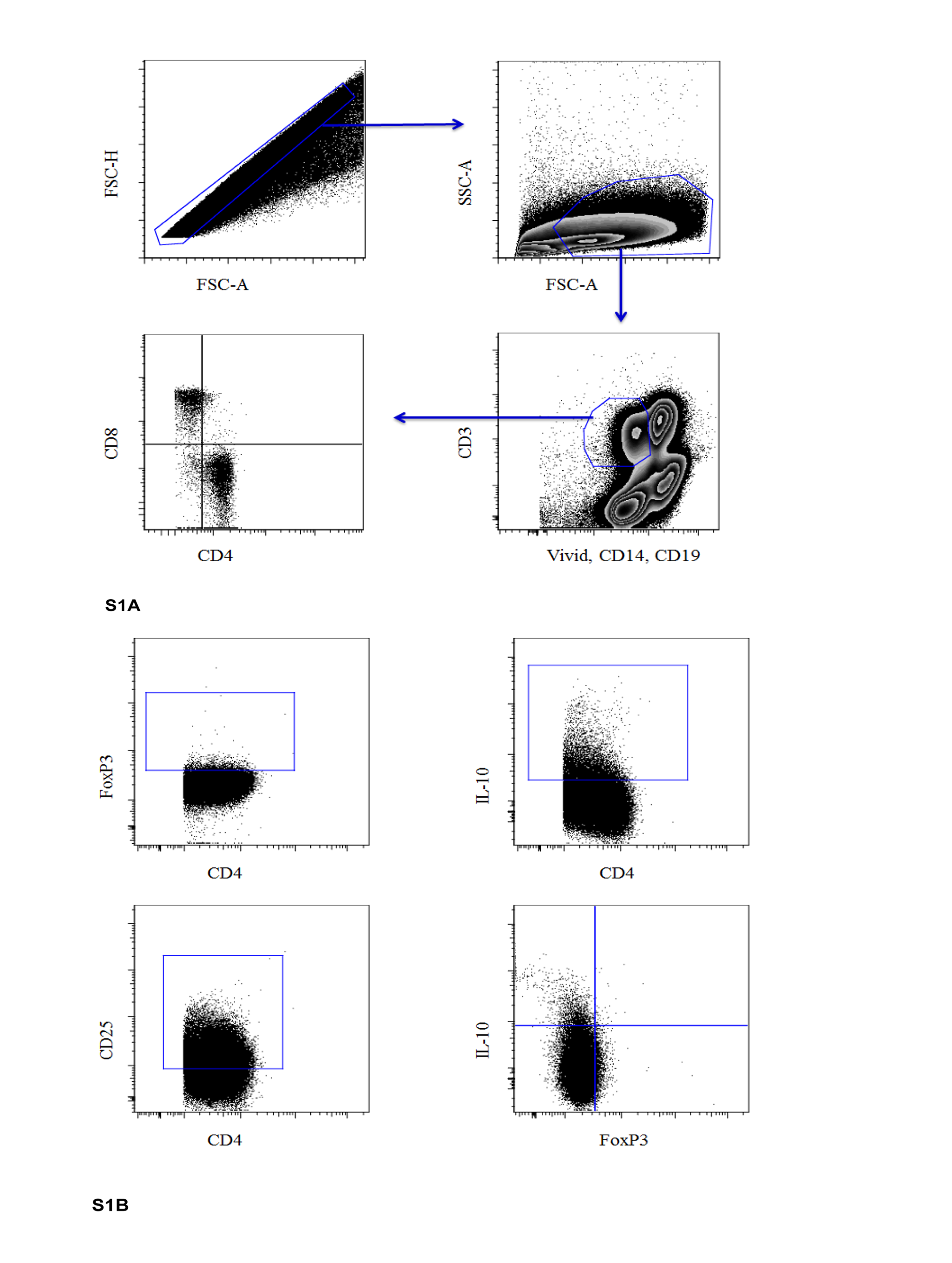

Supplement: Figure S1 — A. Gating strategy for live CD4+CD3+ cells or CD8+CD3+ cells in PBMC. Ungated events were first gated using a forward scatter area (FSC-A) versus height (FSC-H) plot to remove doublets. Subsequently, the events were subjected to a lymphocyte gate by gated through a side scatter (SSC). Subsequently, live CD3+cells were gated by live/dead staining using Vivid (Invitrogen, Life technologies) as a marker for viability and CD14+ or CD19+ events were excluded from analysis using a dump channel. Finally, CD3+ live cells were separated into CD4+ and CD8+. B. Gating strategy for IL-10 and FoxP3 expression in CD4+CD3+ cells or CD8+CD3+ cells. After the gates for each function were created, we used the Boolean gate platform to identify all functions within each cell population using the full array of possible combinations FACS LSR Fortessa as shown here for IL-10 and FoxP3 expression in CD4+ T cells. (TIF) [file pntd.0002773.s001.tif]

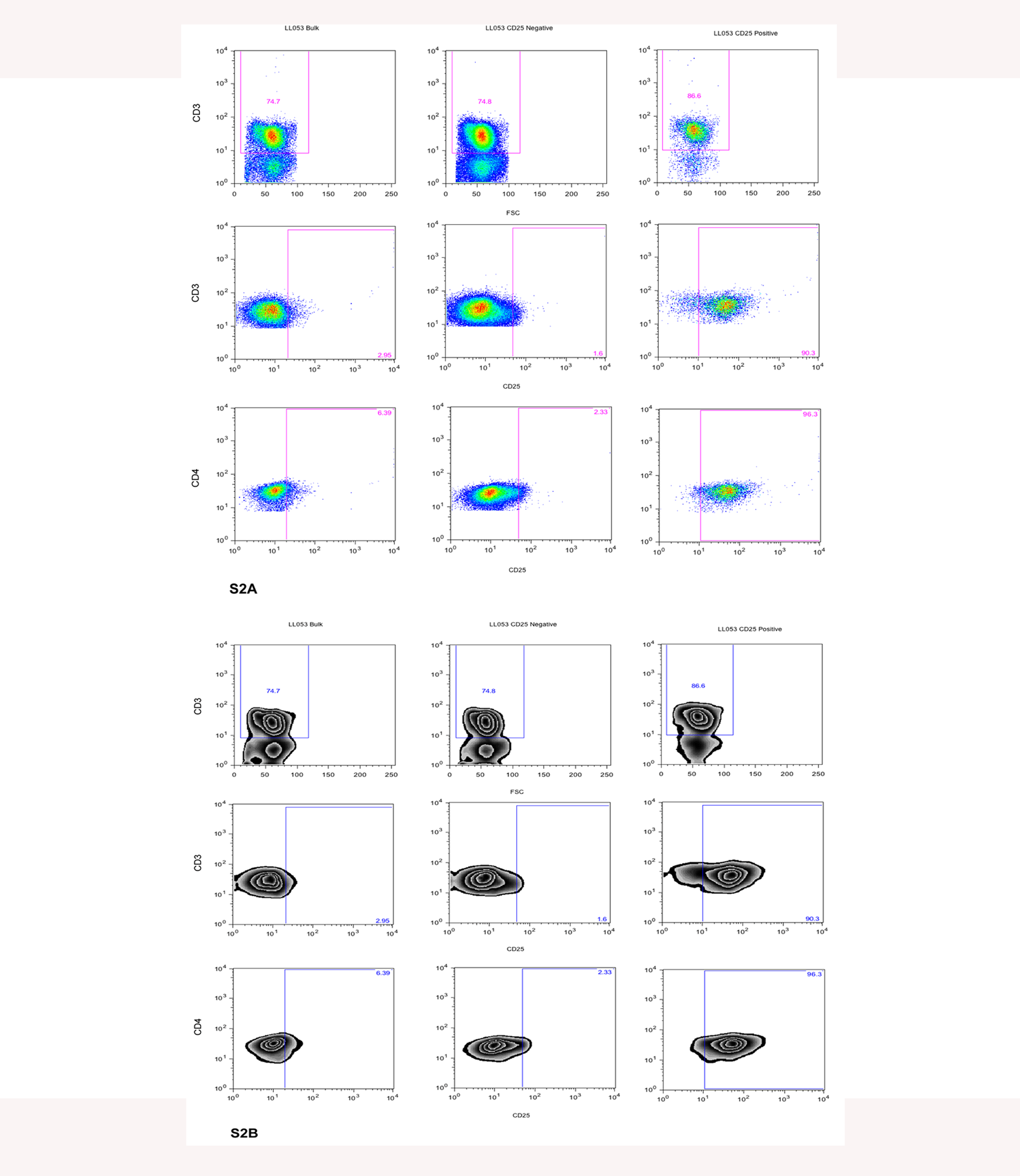

Supplement: Figure S2 — A. Dot plot analysis of bulk (total) PBMC, CD25 depleted and CD25 positive population of a representative LL patient (LL053). After separating the CD25 negative and CD25 positive cell population using Magnetic cell sorter, fractions of each cell population including the bulk (total) PBMC were analysed for their expression of CD3, CD4 and CD25. Here the data are presented in dot plots. B. Zebra plots of bulk (total) PBMC, CD25 depleted and CD25 positive population of a representative LL patient (LL053). After separating the CD25 negative and CD25 positive cell population using Magnetic cell sorter, fractions of each cell population including the bulk (total) PBMC were analysed for their expression of CD3, CD4 and CD25. Here the data are presented in zebra plots. (TIF) [file pntd.0002773.s002.tif]

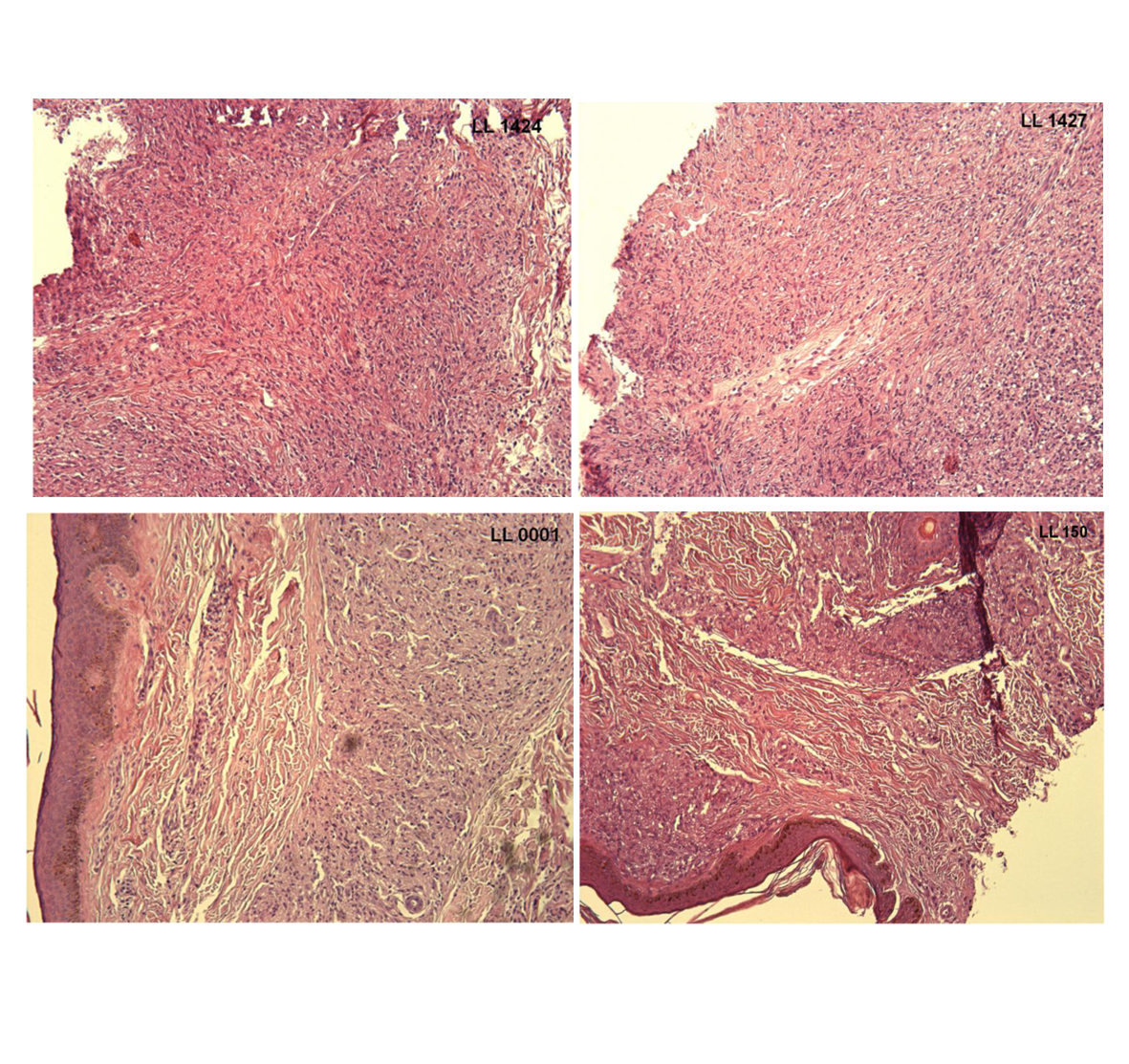

Supplement: Figure S3 — Hematoxylin and Eosin staining of four representative LL patients (original magnification ×100). Tissue sections from paraffin embedded biopsy samples of leprosy patients were stained for H&E. Here images of H&E staining of four representative LL patients are presented. (TIF) [file pntd.0002773.s003.tif]
